# Multi-Organ toxicity demonstration in a functional human *in vitro* system composed of four organs

**DOI:** 10.1038/srep20030

**Published:** 2016-02-03

**Authors:** Carlota Oleaga, Catia Bernabini, Alec S.T. Smith, Balaji Srinivasan, Max Jackson, William McLamb, Vivien Platt, Richard Bridges, Yunqing Cai, Navaneetha Santhanam, Bonnie Berry, Sarah Najjar, Nesar Akanda, Xiufang Guo, Candace Martin, Gail Ekman, Mandy B. Esch, Jessica Langer, Gladys Ouedraogo, Jose Cotovio, Lionel Breton, Michael L. Shuler, James J. Hickman

**Affiliations:** 1 NanoScience Technology Center, University of Central Florida, 12424 Research Parkway Suite 400, Orlando, FL 32828; 2 Department of Biomedical Engineering, Cornell University, 115 and 305 Weill Hall, Ithaca, NY 14853; 3 L’Oreal Research and Innovation, Clark, NJ, 07666/ Aulnay sous Bois, France, 93600; 4 L’Oreal Research and Innovation, Aulnay sous Bois, France

## Abstract

We report on a functional human model to evaluate multi-organ toxicity in a 4-organ system under continuous flow conditions in a serum-free defined medium utilizing a pumpless platform for 14 days. Computer simulations of the platform established flow rates and resultant shear stress within accepted ranges. Viability of the system was demonstrated for 14 days as well as functional activity of cardiac, muscle, neuronal and liver modules. The pharmacological relevance of the integrated modules were evaluated for their response at 7 days to 5 drugs with known side effects after a 48 hour drug treatment regime. The results of all drug treatments were in general agreement with published toxicity results from human and animal data. The presented phenotypic culture model exhibits a multi-organ toxicity response, representing the next generation of *in vitro* systems, and constitutes a step towards an *in vitro* “human-on-a-chip” assay for systemic toxicity screening.

According to the FDA’s Adverse Event Recording System, 2.3 million reports of adverse drug effects were submitted across 6000 registered compounds between 1969 and 2002^1^. During this period, 75 drugs or drug products were removed from the market due to these unpredicted effects, and a further 11 were given special requirements or restrictions^1^. In addition to withdrawn compounds, only 1 in 10 drugs entering clinical trials typically proves efficacious enough to become registered for human treatments^2^, indicating a significant proportion of compounds validated during preclinical screening have unpredicted problems when introduced into living human systems. The high failure rate of drugs at this late stage of development contributes significantly to this increase in cost as well as delays in the development of new and more effective clinical treatments.

The generation and characterization of *in vitro* systems capable of reproducing the functionality of specific human organs in a quantifiable manner is currently a focal point of intensive research and development given the long-standing inadequacies inherent to the use of conventional preclinical techniques for predicting human tissue behavior^3^,^4^,^5^. Such advanced *in vitro* systems are seen by many as the means to streamline current drug development protocols, and thought to generate more informative platforms with which to investigate human tissue physiology and pathology in defined and controllable environments^5^. Current high-throughput systems for drug discovery and toxicology applications rely on indirect measurement of cell health and function, such as biomarkers and RNA expression analysis. The field would benefit from more advanced systems that are low cost and utilize defined media to support multiple cell types under continuous flow conditions with reproducible functional readouts.

Attempts to observe and characterize the interaction between multiple cell types *in vitro* have already been achieved to some degree. For example, co-culture of gut epithelial Caco-2 cells with hepatocytes has been performed using trans-well membranes^6^, and multiple cell types have been successfully maintained by simply establishing all cultures in confined spaces within a single culture well^7^,^8^. However, these examples lack the dynamic flow of nutrients and toxins generated in living systems for extended time periods (>7 days). Microfluidic techniques enable the culturing of cells in compartmentalized chambers with controlled interactions between each chamber to better mimic blood flow^5^. This technology facilitates the metabolism of the drug in one compartment and subsequent convective transfer of the resulting metabolite into other compartments where its effect on different cell types can be evaluated, but generally these systems are utilized for acute toxicity evaluations. In addition, most systems have employed peristaltic or other pump types as well as bulky valves and tubing that increases cost and introduce bubbles and other factors detrimental to system function. These proof-of-concept studies successfully demonstrate the potential, but also some of the drawbacks, for using such microsystems technology to study simple interactions between organs.

Research focused on extending the lifespan of these microfluidic devices has been the main focus of many laboratories to validate body-on-a-chip systems as models for repeated dose or chronic exposure of compounds for efficacy, toxicity and pharmacokinetic studies. Currently, the toxicology models used to study chronic exposures are still animals, and a focus of this research is to switch to the utilization of *in vitro* models. More recently, a two organ model, incorporating liver and skin micro-tissue mimics, has been developed and data presented which validates its ability to maintain these cultures out to 28 days *in vitro*^9^. Although microfluidic systems have also been developed to investigate three^10^ and four^11^ organ interconnectivity and functionality *in vitro*, these studies focused on metabolic function over an acute, 48 hour time period. Data supporting the use of these models for measuring the effects of long-term drug treatment, repeat dose studies or to represent chronic pathological conditions have yet to be presented. It is also noteworthy that the majority of these studies^9^,^10^,^12^ have relied on serum-containing medium as well as biomarker readouts as measurements of cell health and function and the use of perfused systems to feed the culture in the device with an external pump. Given that animal sera contain undefined and variable constituents, their inclusion in multi-organ models for drug screening applications limits the predictive capacity of the developed assay since interaction of the compound with unknown components in the medium cannot be effectively controlled^13^. In addition, biomarker assays only approximates cell or tissue function and are costly to measure and external pumps increase the footprint of the device and the ultimate price for any marketed system or services.

This study details the development and characterization of a low-cost gravity driven flow system^14^ for maintaining viable and functional human cardiac, liver, skeletal muscle and neuronal cultures within a common defined medium for two weeks. These cell types were chosen to provide insight into important metabolic and functional changes in human tissues in response to challenge with compounds with well-defined toxicological properties. The presented data demonstrates the survival and continued functionality of all 4 human cell types over a 14 day culture period under flow in this microfluidic system to demonstrate the ability to reach chronic timeframe, as well as their response to therapeutic challenge from five compounds at 7 days for acute responses. The cultures were evaluated *in situ* and *ex situ* to show both the ability to carry out non-invasive analysis or the capability to perform more extensive testing after disassembly. This low cost platform was self-contained and allowed for continuous circulation of the blood surrogate with periodic medium supplementation and analyte analysis and represents the next generation of organ-on-a-chip or body-on-a-chip systems for both acute and chronic studies.

## Results

### Device design and gravitational flow simulations

**Figure 1:**
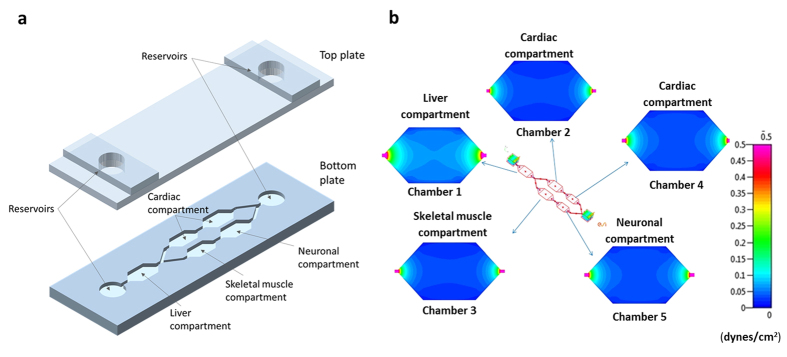
(**a**) Schematic view of the microfluidic platform showing the different cell compartments. The system contained two holders for the separate culture devices. Total fluid volume was approximately 4 mL between the chambers and reservoirs. The size of the culture compartments were 35.8 × 18.4 × 0.3 mm for Chambers 1, 2, 3 and 29.8 × 15.4 × 0.7 mm for Chambers 4, 5. The connecting channel dimensions were 5.7 × 1 × 0.3 mm. (**b**) Shear stress distribution in each compartment of the system.

A schematic overview of the system, indicating the location of each cell compartment within the fluidic platform, is shown in Fig. 1a. Although we show specific tissues in these chambers the system is modular and reconfigurable in that other organ modules can be substituted in the device. A gravity-induced flow was enabled by utilizing a rocker platform to generate shear stress on the cellular monolayers within accepted ranges^14^,^15^. Flow rate and shear stress on the cells were determined by the choice of tilt angle, periodicity of the tilting action and the cross sectional area of the channels and chambers. Air bubble formation within the device was greatly reduced as they were prevented from entering the system due to buoyancy, and communication between chambers ensured equal oxygen and nutrient distribution throughout the platform. Figure 1b indicates the shear stress distribution in each chamber of the system when the gravity-induced flow was applied, calculated utilizing the computational fluid dynamics program CFD-ACE^+^. The results from the simulations were used to determine the effects of tilt angle and time period of oscillation of the rocker platform to program the flow rate in the system and to keep shear rates within physiological ranges (more detail in supplement files). For example, a tilt angle of 8° with two oscillations per minute gives a maximum shear stress of 0.25 dynes/cm^2^, which is within the physiological values seen for internal organs such as the liver^15^.

### Cell survival and functional evaluation in flow conditions

**Figure 2:**
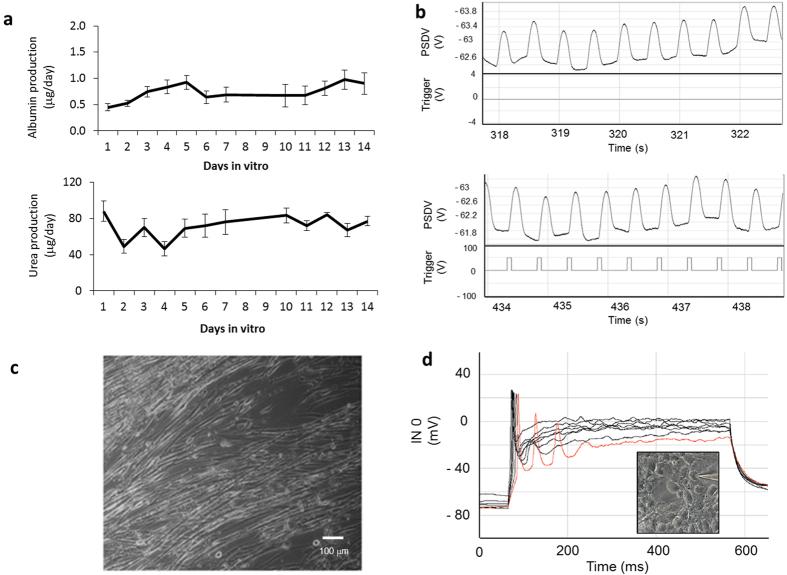
Functional data recorded for the different cell types after 14 days in the system under flow. (**a**) Albumin (top) and urea (bottom) production by HepG2/C3A cells. Data is presented as mean ± standard error of the mean. (**b**) Top: spontaneous contractile activity of cardiomyocytes on microscale silicon cantilevers after 14 DIV. Bottom: controlled contractions of cardiomyocytes on microscale silicon cantilevers after 14 DIV in response to broad field electrical stimulation (2 Hz). **(c)** Skeletal muscle contractility was assessed by video analysis (Supplementary materials). (**d**) Electrophysiological action potentials in motoneurons. Inset: image of patched cell.

Each cell type is cultured on a “module,” which is inserted into the system. These modules allow measurement of not only metabolic but electrical and mechanical function. Data obtained from cell viability and functionality assessment after 14 days of co-culture under flow in the system are shown in Fig. 2. Human serum albumin (HSA) production and urea production by human liver cells were assessed throughout the culture period by analyzing samples of conditioned serum-free medium removed from the platform reservoirs each day (30% of the total volume). Figure 2a indicates stable levels of albumin (p = 0.17) and urea (p = 0.88) for liver function that indicate no significant changes during the experiments when no drugs are added. The same volume of fresh medium replaced the conditioned medium removed to replenish nutrients. Cardiomyocyte *in situ* measurement function was evaluated by measuring the amount of deflection of microfabricated cantilevers from contracting cell monolayers grown on these BioMEMs devices^16^. Figure 2b shows cardiomyocyte spontaneous contractile activity after 14 days in the system (top) as well as controlled cardiomyocyte contractility in response to broad-field electrical stimulation pulses at 2 Hz (bottom). Skeletal muscle cultures were assessed using video analysis and found to contract in culture after 14 days under broad-field electrical stimulation (Fig. 2c, video in Supplementary Material). Neurons were analyzed at day 14 using whole cell patch clamp electrophysiology and were found to possess strong inward sodium and outward rectifying potassium currents. All cells investigated were found to be capable of generating action potentials when stimulated after 14 days (Fig. 2d), and no decrease in cell number of more than 29 ± 17% was observed after 2 weeks in co-culture. The 4-organ co-culture under flow did not appear to affect the cell viability significantly of any organ mimic during the experiment. These observations together demonstrate that the base system (no drugs added) operates stably for at least two weeks in a common serum-free medium. Functional measurements of electrical and mechanical (force) as well as video analysis, both *in situ* and *ex situ*, are clearly possible for chronic experiments.

### Multi-organ toxicological studies

**Figure 3:**
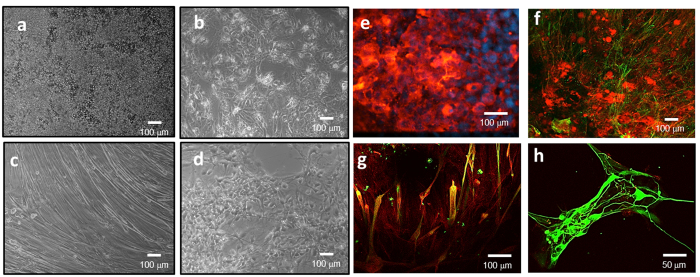
Bright field microscopy images (10×) of (**a**) HepG2/C3A, (**b**) iPSC derived human cardiomyocytes, (**c**) skeletal muscle cells and (**d**) neurons after 7 days in co-culture in the microfluidic system, in serum free medium and under flow conditions. Immunocytochemical staining of (**e**) hepatocytes stained for albumin (red) and DAPI (blue), (**f**) iPSC derived cardiomyocytes stained for troponin (green) and actin (red), (**g**) skeletal muscle stained for myosin heavy chain (green) and actin (red) and (**h**) neurons stained for neurofilament (green) and actin (red) after 7 days in co-culture in the system. (scale bars a–g = 100 μm; h = 50 μm).

The compounds chosen for the study were Doxorubicin (DOX) 5 μM, Atorvastatin (ATR) 100 μM, Valproic acid (VPA) 2 mM, Acetaminophen (APAP) 5 mM, and the control compound, N-Acetyl-m-aminophenol (AMAP) 5 mM. A compilation of the results obtained from the systemic toxicity study of each drug in the 4 organ system are compared to literature reports in Table 1. The toxicity trials were initiated after 5 days under flow in the pumpless system and lasted for 48 hours. Drugs were administered through the reservoir closest to the liver. The cultures were compared to untreated controls at 7 days in the flow system for both function and viability. In the control 7 day cultures, bright field microscopy images indicated normal morphology of the different cell types in the microfluidic platform under flow are shown in Fig. 3a–d. Examples of cell survival and expected phenotype after 7 days in co-culture were also confirmed by immunocytochemical staining, as shown in Fig. 3e–h.

#### Doxorubicin (DOX)

Cardiac cell viability is compromised *in vitro* when incubating with DOX due to induction of apoptotic pathways and increased reactive oxygen species (ROS) production^17^. The dose used in this report (5 μM) is higher than what is typically detected in patient plasma concentrations (0.5–1 μM) but consistent with what it is found in mitochondria (typically 100 times more concentrated than plasma levels)^17^. As the metabolism of DOX in the body is predominantly executed by the liver, high amounts of oxidative stress are generated in this organ, leading to hepatotoxicity as another side-effect of DOX treatment. 40% of patients undergoing DOX treatment show hepatotoxicity due to reduced cellular regeneration, an increase in lipid peroxidation, a decrease in glutathione (GSH) levels to counteract oxidation, DNA damage and apoptosis induction through the release of cytochrome c from the mitochondria^17^.

**Figure 4:**
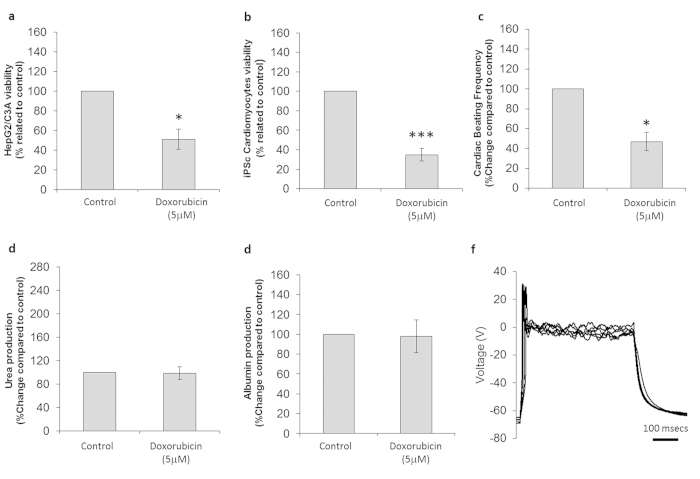
Cytotoxic effects on cells following treatment with doxorubicin. (**a**) HepG2/C3A viability assay results. (**b**) Cardiomyocyte viability assay results. (**c**) Comparison of drug-treated and untreated cardiomyocyte beating frequency. (**d**) Urea production in controls and drug-treated hepatocytes. (**e**) Albumin production in control and drug-treated hepatocytes. (**f**) Representative electrophysiology recording of a drug-treated neuron. All presented data is displayed as mean ± standard error of the mean. (*p < 0.05/ ***p < 0.001 compared to control).

The overall cytotoxic effects of DOX are summarized in Table 1, where cell viability and cell functionality data obtained with the microfluidic system are compared to general toxic effects reported in the literature. MTT assays of the DOX treated system indicated a decrease in viability of both hepatocytes and cardiomyocytes of 49 ± 10% (p = 0.012) (Fig. 4a) and 65 ± 6% (p = 0.0004)(Fig. 4b) respectively. The toxic effect on cardiomyocytes was also shown functionally by a 47 ± 9% decrease in beat frequency (p = 0.049) (Fig. 4c). No effects on albumin (p = 0.48) and urea (p = 0.48) production were observed (Fig. 4d–e). Whereas muscle morphology was not affected, the contractile activity was shown to cease in 60% of the cultures. Neuronal electrophysiology showed no changes in ion currents or firing of stimulated action potentials, which corresponds to healthy, functional neurons (Fig. 4f). In addition, action potential peak voltage (p = 0.24), resting membrane potential (p = 0.37) and peak ionic currents (Na^+^ p = 0.34, K^+^ p = 0.32) were not significantly different between treated and untreated control. However, there was a loss of repetitive (p = 0.008) and spontaneous (p = 0.082) firing ability. The neuronal morphology study also indicated a 33 ± 18% cell loss compared to the control condition (p = 0.088). These data show multiple effects of doxorubicin on various organs in the system.

#### Atorvastatin (ATR)

Statins are reported to induce different grades of myotoxic effects in a significant number of patients^18^,^19^. The ATR concentration used in this study (100 μM) is within the range for human plasma levels (13–125 μM)^20^,^21^ and the overall cytotoxic effects found are reported in Table 1.

**Figure 5:**
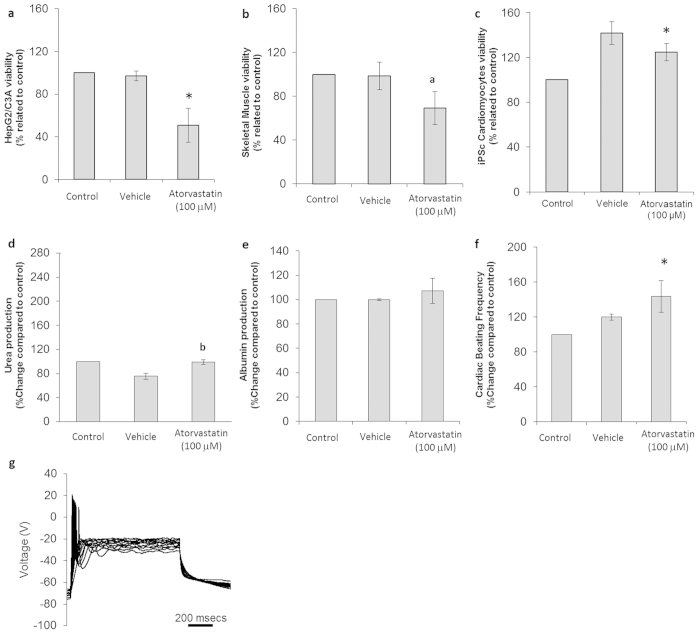
Cytotoxic effects on cells following treatment with atorvastatin. (**a**) HepG2/C3A viability assay results. (**b**) Skeletal muscle viability assay results. (**c**) Cardiomyocyte viability assay results. (**d**) Urea production in controls and drug-treated hepatocytes. (**e**) Albumin production in control and drug-treated hepatocytes. (**f**) Comparison of drug-treated and untreated cardiomyocyte beating frequency. (**g**) Representative electrophysiological recording of a drug-treated neuron. All presented data is displayed as mean ± standard error of the mean. (b p ≤ 0.1/a p ≤ 0.08/*p ≤ 0.05 compared to control).

ATR treatment in the system resulted in a 50 ± 16% decrease of hepatocyte viability (p = 0.017) (Fig. 5a), and a 30 ± 15% decrease in skeletal muscle viability (p = 0.068) (Fig. 5b), compared to the vehicle control (DMSO), and muscle cellular contractility was shown to cease in 50% of the cultures. ATR increased hepatocyte urea production by 24 ± 4% (p = 0.095) (Fig. 5d), while no change in albumin production was observed (p = 0.39) (Fig. 5e). A significant increase in cardiac beat frequency (43 ± 16% from 0.9 ± 0.2 Hz) (p = 0.035) after 7 days in co-culture was observed compared to the vehicle (Fig. 5f). Along with functionality, cardiac viability also increased (24 ± 8%) (p = 0.015) compared to the control (Fig. 5c). The neuronal electrophysiology analysis, although showing typical ion currents and stimulated firing APs, indicated altered ability to fire APs repetitively (p = 0.001) and spontaneously (p = 0.082) as well as changes in it’s peak voltage (p = 0.071) and resting membrane potential (p = 0.030) (Fig. 5g). The neuronal morphological analysis revealed a 47 ± 14% cell loss compared to the control condition after a 48 h treatment (p = 0.030).

#### Valproic Acid (VPA)

At therapeutic dosages, VPA is detected in the range of 300–700 μM in patient plasma^22^, while concentrations around 2 mM are known to be cytotoxic *in vitro*^23^. Clinical data correlates VPA with idiosyncratic hepatotoxicity; liver failure has been reported previously, but at a very low rate of approximately 1 in 15,000. However, children under 2 years old undergoing polytherapies do suffer from a higher rate of fatal hepatotoxicity in response to VPA treatment^22^,^23^,^24^. VPA is rapidly metabolized to various pharmacologically active metabolites, *trans*-2-en-valproate being one of the most active products^22^. A concentration of 2 mM was tested in the 4-organ platform. The overall results of the cytotoxicity study for VPA are reported in Table 1.

**Figure 6:**
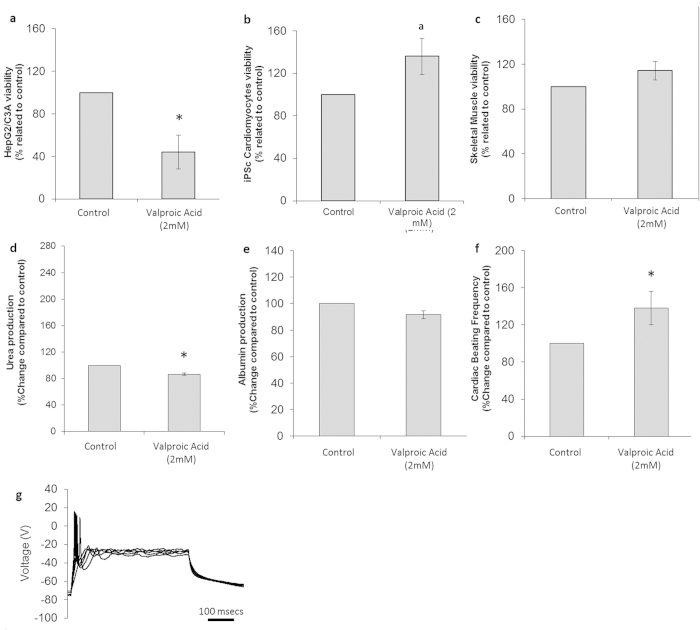
Cytotoxic effects on cells following treatment with valproic acid. (**a**) HepG2/C3A viability assay results. (**b**) Cardiomyocytes viability assay results. (**c**) Skeletal muscle viability assay results. (**d**) Urea production in controls and drug-treated hepatocytes. (**e**) Albumin production in control and drug-treated hepatocytes. (**f**) Comparison of drug-treated and untreated cardiomyocyte beating frequency. (**g**) Representative electrophysiological recording of a drug-treated neuron. All presented data is displayed as mean ± standard error of the mean. (a p < 0.08/*p < 0.05 compared to control).

A decrease of 56 ± 16% (p = 0.020) for hepatocyte viability following drug treatment in the system was observed (Fig. 6a), while hepatic metabolic functionality was slightly affected (Fig. 6d,e) with a 13 ± 2% (p = 0.015) decrease in urea production but no changes in HSA levels (p = 0.15). Treatment with VPA resulted in a cardio-protective effect, with an increase of both cardiomyocyte viability (36 ± 18%) (p = 0.051) and beat frequency (38 ± 18% from 0.9 ± 0.2 Hz) (p = 0.039) after 7 days in co-culture (Fig. 6b,f). No significant effect on skeletal muscle viability was observed (Fig. 6c)) but there was a loss of contractile activity in 76% of the cultures. The neuronal electrophysiology analysis indicated no changes compared to the control (Fig. 6g) (p = 0.76).

#### Acetaminophen (APAP)

The therapeutic, and safe, plasma concentration for APAP is in the low micromolar range. This is reached when taking 500–1000 mg orally three times a day, with a maximum daily dose of 4 g^25^,^26^,^27^. Toxic APAP plasma concentrations depend on the half-life of the compound in humans, being around 2 mM the first two hours after ingestion, and falling to less than 50 μM after 24 hours^26^,^28^. A dosage of 5 mM over 48 hours in the 4-organ model indicated a decrease in liver cell viability under flow conditions. The overall cytotoxic effects of APAP are summarized in Table 1.

**Figure 7:**
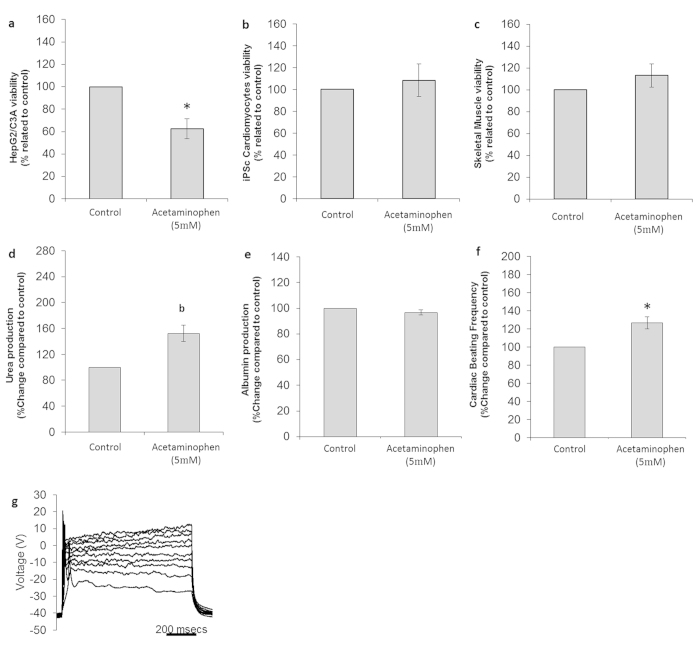
Cytotoxic effects on cells following treatment with acetaminophen. (**a**) HEPG2/C3A viability assay results. (**b**) Cardiomyocyte viability assay results. (**c**) Skeletal muscle viability assay results. (**d**) Urea production in controls and drug-treated hepatocytes **(e)** Albumin production in control and drug-treated hepatocytes. (**f**) Comparison of drug-treated and untreated cardiomyocyte beating frequency. (**g**) Representative electrophysiological recording of a drug-treated neuron. All presented data is displayed as mean ± standard error of the mean. (b p < 0.1/*p < 0.05 compared to control).

APAP treatment resulted in a 37 ± 9% decrease in hepatocyte viability (p = 0.012) (Fig. 7a) and while albumin production was not affected (p = 0.25) (Fig. 7e), urea production increased by 52 ± 13% (p = 0.095) (Fig. 7d). Viability of cardiomyocytes (Fig. 7b) and skeletal muscle (p = 0.17) (Fig. 7c) were not significantly affected by treatment with APAP. However, there was a 33% decrease in the number of skeletal muscle cultures that contracted and an increase in the beating frequency of the cardiomyocytes by 27 ± 9% (p = 0.017) (Fig. 7f). The electrophysiology of the neurons indicates the loss of repetitive (p = 0.0004) and spontaneous (p = 0.082) firing ability, but no changes in ion currents or the firing of stimulated action potentials, indicating healthy neurons (Fig. 7g). A reduction of neuronal cell numbers after the 48 h APAP treatment was 50 ± 18% (p = 0.036) and was contrary to expectations.

#### N-Acetyl-m-aminophenol (AMAP)

**Figure 8:**
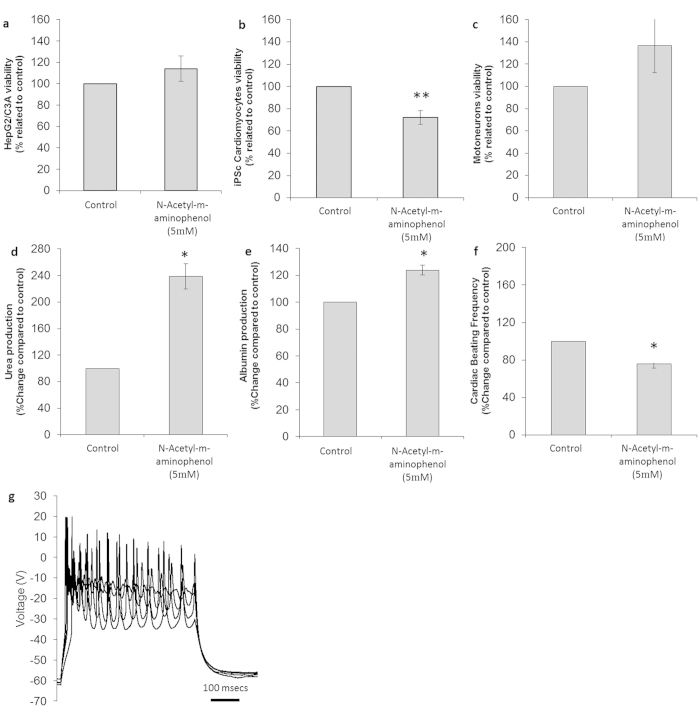
Cytotoxic effects on cells following treatment with N-Acetyl-m-aminophenol. (**a**) HEPG2/C3A viability assay results. (**b**) Cardiomyocyte viability assay results. (**c**) Neuronal viability assay results. (**d**) Urea production in controls and drug-treated hepatocytes. **(e)** Albumin production in control and drug-treated hepatocytes. (**f**) Comparison of drug-treated and untreated cardiomyocyte beating frequency. (**g**) Representative electrophysiological recording of a drug-treated neuron. All presented data is displayed as mean ± standard error of the mean. (*p < 0.05 **p < 0.01 compared to control).

Like APAP, AMAP exerts analgesic and antipyretic properties, but contrary to APAP, AMAP does not induce hepatotoxicity. AMAP was used as a negative control for APAP in this study, and was applied at the same concentration and conditions. The cytotoxic effects of AMAP are summarized in Table 1. No significant effects were observed in the system with AMAP treatment in viability data for hepatocytes (p = 0.15) and neurons (p = 0.19) (Fig. 8a,c). While it did not affect hepatic viability, as seen with APAP, there was a net increase in urea production (p = 0.02) (Fig. 8d) and albumin (p = 0.032) (Fig. 8e). The neuronal electrophysiology showed no significant changes compared to the control (p = 0.19) (Fig. 8g), and while there was some increase in cell numbers, this was not a significant change after the 48 hour treatment (p = 0.19). Muscle viability and contractile ability were not affected (p = 0.46). However, cardiomyocyte viability was reduced by 28 ± 6% (p = 0.005), and beat frequency dropped by 25 ± 4% (p = 0.012) (Fig. 8b,f). These results support the conclusion that AMAP is mildly cardiotoxic in this four organ system. It is unclear whether AMAP induced cardiac toxicity exists *in vivo* as it is most commonly used as a liver toxicity control in studies where no cardiac data is collected.

## Discussion

This is the first multi-organ human system to use a pumpless platform with electrical and mechanical readouts to assess organ component health. The use of functional readouts beyond metabolic biomarkers opens new opportunities for these microphysiological systems to give deeper insight into physiological responses as well as lower cost and allow for chronic non-invasive monitoring of these systems. The other key developments with this platforms being the integration of the four organ systems together under continuous recirculating flow in a serum-free medium, which has also not previously been demonstrated. Given that animal sera contain undefined and variable constituents, their inclusion in models for drug screening applications limits the predictive capacity of the assay since interaction of the compound with unknown components in the medium cannot be effectively controlled^13^ and has been advocated for in multiple studies^10^,^14^,^29^,^30^. Serum-free media also promotes the maintenance of differentiated cellular function^31^ which is important for these *in vitro* models, but is often difficult to attain with multiple cell types for long periods in a common medium. The platform utilized a pumpless system where circulation was achieved through gravity driven flow^14^. This unique pumping method avoids the need for external pumps, tubing and valves resulting in a system of decreased complexity, which should decrease cost and improve operability (e.g. reduce problems due to gas bubble entrapment). The system was shown to be viable and functional for up to 14 days under continuous flow. Single organ systems have been reported over the last 15 years which mimic the functionality of a wide variety of tissues, and while they are important (see Esch^3^ and references contained therein), their *in vivo* counterparts rarely act in isolation. Here, we demonstrate a system that allows one to determine if a single drug affects multiple organs simultaneously.

The organ chips were distributed in separate compartmentalized chambers, each interconnected to one or two other chambers though microfluidic channels. Human cell types of the four organs were represented with cell lines, primary cells and cells derived from iPSc allowing the exchange of metabolites and signal molecules. Functional evaluation of the organs after pharmacological exposure for 48 hours was achieved by measuring cardiac beat frequency, muscle contractibility, neuronal electrophysiology as well as liver albumin and urea production. The results were compared to human clinical data or other data from the literature to provide initial validation of the presented system as a model for accurately predicting multi-organ toxicity in humans. The utilization of functional measures of organ status allows for a decreased reliance on biomarkers as indicators of cell health, but also enables noninvasive monitoring of organ health and function for both acute and, *more importantly*, chronic experiments in the future.

The four human organs or tissues represented in this platform are common targets in drug development and toxicity but have only indicated multi-organ toxicity in *in vivo* results previously. The liver is a key processor of drug metabolites and regulator of drug half-life. It is a common target of unpredicted harmful side-effects caused by drug or metabolite exposure^32^,^33^. The second most important tissue is the ventricular myocardium as effective cardiac modeling is the subject of intense research since 40–70% of all drug attritions during development are due to arrhythmogenic properties^34^,^35^. Adequate modeling of cardiac rhythm generation is essential for any multi-organ drug screening platform. The third and fourth organs represented in the platform are muscle and the nervous system. Skeletal muscle is responsible for significant levels of glucose storage in the body and accounts for a substantial proportion of overall energy expenditure. It is a common target for unpredicted compound side effects, which can have a significant impact on patient quality of life (through induction of tremors, spasms, atrophy, and muscular pain). The neuronal compartment represents a particularly sensitive cellular system and a means to introduce the ability to monitor non-fatal central nervous system effects into this multi-organ platform by measuring electrophysiological function.

Immunocytochemical staining of specific markers for each tissue highlight the successful maintenance of all cell types in this dynamic long-term culture system. The functional readouts collected following 7 or 14 days in culture include albumin and urea production levels from the liver fraction, observation of cardiac electrical properties, measurement of contractile activity in the cardiac and the skeletal muscle fractions and recordings of action potential firing activity in the cultured neurons. The correlation of observed results from this system with human *in vivo* data as shown in Table 1 validates the platform as a novel tool for studying and eventually predicting drug efficacy and toxicity. The concentration analyzed for each compound was selected to produce modest toxic effects within 48 hours in the specific organs, and the dosages were based on the available literature. Doxorubicin (DOX) is a widely used chemotherapeutic that impairs replication and transcription, but has a strict dose limit due to its established cardiotoxic side effects^36^. The cardiotoxicity experienced by patients treated with DOX can be due to acute or chronic (cumulative) dosages^17^,^36^. A 48 hour treatment with 5 μM DOX in the 4-organ system, representing an acute dosage, generated a 65 ± 6% loss in the cardiac cell population compared to untreated controls. Among the viable cells, the system indicated a 47 ± 9% decrease in the spontaneous beat frequency, which is in line with previously reported data using other *in vitro* cardiac culture systems^37^. The mechanism of this toxicity appears to be multifactorial^17^,^36^,^38^,^39^. The generation of ROS, and the degradation of DOX to its toxic metabolite, doxorubicinol, are the principal causes of the apoptotic side effects^17^. Doxorubicinol is a potent cardiotoxic agent that inhibits cardiac function and the ATPase activity of the sarcolemma, mitochondria, and sarcoplasmic reticulum^38^,^39^.

Another known side effect of DOX treatment involves hepatotoxicity, which was observed in our system by a decrease in viability (49 ± 10%), but not in hepatic metabolism in the first 48 hours after drug exposure. The depletion of glutathione levels, and thus antioxidant levels, is one of several mechanisms involved in DOX induced hepatotoxicity^40^. The neuronal fraction of the 4-organ platform was significantly affected by the DOX treatment (33 ± 13% cell loss), however neurons continued to fire action potentials, although the repetitive and spontaneous APs were not observed after treatment. This is consistent with a report by Aquilano and coworkers where neuron viability dropped 25% when exposed to 1uM DOX for 24 hours^41^. With respect to the skeletal muscle fraction, DOX is known to induce a toxic effect at physiological concentrations through a Ca^2+^ handling impairment and an increase in oxidative stress, leading to muscle weakness and fatigue, but not atrophy^42^,^43^. The DOX concentration assessed in this report slightly affected muscle viability and reduced contractile activity in 60% of the cultures, supporting the literature reports of force reduction.

Atorvastatin (ATR) is a commonly prescribed statin used for cholesterol homeostasis. A 48 hour treatment with ATR (100 μM) in the 4-organ system induced myotoxicity, reducing cell viability by 30 ± 15%, and reduced myotube contraction events (50%). Statin induced myotoxicity is reported to induce different grades of myopathy in a significant part of the population, ranging from mild myalgia to fatal rhabdomyolysis^19^. ATR is metabolized by the liver (CYP3A4 is the main enzyme participating), and the reduction of its metabolism by inhibiting CYP3A4 activity triggers this toxicity^18^. A 48 hour incubation of ATR (100 μM) in the liver mono-culture condition induced CYP3A4 activity by 12-fold (data not shown). Interestingly, ATR myotoxicity in the co-culture is not decreased by the presence of the liver. Statins rarely cause clinically significant liver injury, although asymptomatic elevation in aminotransferase levels is observed in 1–3% of patients^44^,^45^,^46^. ATR-related hepatotoxicity has been associated with a mixed pattern of liver injury, typically occurring several months after the initiation of the medication. Hepatotoxicity was detected in the 4-organ platform and characterized as a reduction in liver cell viability by 50 ± 16%.

In the cardiac compartment, no reduction was observed in cell viability following ATR treatment, and a 43 ± 16% increase in beat frequency was recorded. ATR is known to play a protective role in induced cardiotoxicity^47^, and has been used as a negative control in cardiotoxicity studies^37^; the results obtained in this study are consistent with the previously published data. A significant reduction of 47 ± 16% in cell viability was observed in the neuronal fraction upon ATR incubation, but regular AP activity was recorded with good sodium and potassium currents, although no repetitively firing cells were observed. Studies using animal models and various cell types indicate some inconsistencies regarding ATR toxicity in the nervous system. Previous studies have suggested a neuroprotective effect at low concentrations (100 nM to 1 μM), but neurotoxic events in the micromolar range^48^,^49^. ATR is able to cross the blood brain barrier due to its lipophilic character^50^, and inhibit brain cholesterol biosynthesis with possible consequences for synaptic cholesterol homeostasis^51^. Results obtained in this study using a relatively high ATR concentration of 100 μM could correlate with the observed toxicity in previous *in vitro* and *in vivo* studies^49^.

Valproic acid (VPA) is a first-line anticonvulsive agent and is also used as a mood stabilizer and other indications are currently being studied through clinical trials^24^. VPA potentiates the GABA inhibitory effect by blocking its degradation, and also by the blocking of ion channels. The incubation with 2 mM VPA in the 4-organ system affected the liver fraction by reducing viability by 56 ± 16% and decreasing urea production by 13 ± 2%. Other *in vitro*^23^,^52^,^53^ and *in vivo*^54^ studies using hepatoma cell lines have reported cytotoxicity with VPA (in the millimolar range) in a dose-dependent manner. Hepatocellular damage after VPA treatment has also previously been reported in mice^54^.

VPA incubation in this system exhibited a protective effect on neurons and maintained the electrophysiological parameters of the cells (in terms of spontaneous firing behavior) throughout the experiment compared to untreated controls. This finding corresponds with previous references that report a general neuroprotective effect of VPA^22^,^24^. VPA has been identified as a drug candidate for spinal muscular atrophy or being motoneuron protective^55^. VPA exerted a protective effect in the cultured cardiac cells, with a 36 ± 18% increase in viability, and a 38 ± 18% increase in beat frequency. This result is consistent with previous data on the induction of VPA mediated cardioprotection during cardiac remodeling and myocardial function after infarction in rats^56^.

Acetaminophen is a derivative of acetanilide and widely used as an over-the-counter analgesic and antipyretic^25^,^57^. However, overdoses with APAP cause acute liver failure in humans^58^. It’s metabolism by the liver leads to the oxidized N-acetyl-*p*-benzoquinone imide (NAPQI) metabolite through a secondary pathway that involves the cytochrome p450 system. NAPQI is conjugated with glutathione (GSH) and finalizes its cycle with kidney elimination. In a GSH depletion scenario (APAP overdose or specific p450 enzyme induction), non-conjugated NAPQI disrupts cellular homeostasis, leading to hepatotoxicity^25^,^27^,^57^. Interestingly, APAPs toxicity is species specific; hamsters, mice and humans are sensitive to acetaminophen overdoses, whereas rat, rabbit and guinea pig are not^57^, highlighting the importance of using human cell types for effective preclinical drug toxicity screening. The dose of 5 mM, chosen to mimic an overdose serum level, compromised liver function by reducing HepG2/C3A viability by 37 ± 9% and increasing urea production by 52 ± 13%. Ma and coworkers showed previously a 60% reduction in HepG2 viability inside a microfluidic system with double the concentration of APAP described in this manuscript^59^.

Recent laboratory and pre-clinical studies correlate beneficial effects of APAP on skeletal muscle, cardiac and neuronal function^25^. With this regard, cardiac viability and functionality was preserved (even improved) upon APAP treatment. Concentrations in the millimolar range have been used previously in other cardiac *in vitro* assays as negative controls for cardiotoxicity^37^. This general protective characteristic of APAP could be due to the antioxidant property that APAP exerts in the therapeutic range as suggested by Blough and Wu^25^. However, contrary to results by Blough and coworkers, neurons lost some viability as well as the ability to spontaneously and repetitively fire and muscle exhibited a reduction in contraction for 37% of the cultures. Since our exploratory studies focused on device development rather than specific toxicological questions, this result will need to be verified through many more repeats.

Finally, AMAP, which is a regioisomer of APAP, with the hydroxyl group located at the meta-, instead of in the para- position was treated as a non-toxic control. AMAP metabolites lead to reduced glutathione depletion in comparison with APAP^60^. The lack of liver toxicity induced by AMAP however, has generated some controversy in the literature^61^,^62^. The results obtained from the 4 human organ system support the innocuous characteristics of AMAP for the liver, when compared to hepatotoxic APAP. Interestingly, some liver functionality appeared to be affected as a significant increase in urea production was observed (138 ± 19%) upon drug incubation. Urea production was also up-regulated by its regioisomer APAP, although in a less acerbated manner (52 ± 13%). An increase in albumin production was also observed (23 ± 4%). Of particular note, AMAP was the only drug that modified albumin secretion in the first 48 h. Evidence for neuroprotective activity in this compound was supplemented by analysis of the neuron fraction, which indicate no change in viability and conserved the repetitive and spontaneous AP firing.

Unlike APAP, AMAP was found to exert a moderate damaging effect on cardiac cells (30% reduction in viability and beating frequency). However, no published data regarding AMAP and cardiomyocytes was found to compare with this observation. This finding suggests studies on AMAP induced cardiotoxicity may be fruitful, and provides an example of how this 4-organ *in vitro* system represents a valuable tool for identifying potential toxic interactions in off-target tissues. Interestingly, different toxicities are sometimes observed with regioisomer molecules, and in this case the hydroxyl located in the *para*- position induces hepatotoxicity and exerts cardioprotective benefits, while the *meta* regioisomer gives the opposite effect and benefits the hepatic fraction and induces some toxicity in cardiac cells.

It should be noted that the neuronal results for the tested compounds were obtained with a mixed population of neurons with some glia and in cases where the overall number of neurons decreased, but the functional electrophysiological properties were stable, it could be that non-neuronal cells were targeted. More interesting is the loss of repetitive firing with some drugs as it’s a subtle change and could be linked to impairment of neuronal communication as opposed to loss of cellular function that is normally measured. In addition, the functional data roughly tracked with the viability data for all cells in the system, but the functional measurement appeared to be more sensitive to toxic evaluation than the assay for cell death. The system described here is clearly a prototype and many improvements are possible. These include the addition of other biological components such as barrier tissues (e.g., skin, GI tract, blood brain barrier) and other organ systems (e.g. kidney, lung, pancreas). The basic format and pumpless system can be expanded to accommodate the inclusion of such systems given its modular and reconfigurable nature. The creation of a common serum free medium that preserves essential functionality is a challenge, but we have preliminary experiments demonstrating that we can maintain at least seven different organs in a common serum free medium. We believe that this prototype system forms a solid basis for more complex and realistic human models for both chronic and acute drug testing.

## Conclusions

We demonstrate here, for the first time, a four organ system with continuous recirculation of a serum free medium that mimics human response to five different drugs for at least 14 days. This system measures electrical and mechanical response to various drugs as well as metabolic response. The design uses a pumpless system which is simple, mechanically reliable, and low cost. The system is modular, reconfigurable, and the organ systems tested (liver, cardiac, neuronal, and muscle) represent the most important organs in terms side effects leading to drug candidate failure. The results obtained from this work indicate that this human 4-organ *in vitro* system is a viable tool to study: (i) organ to organ communication, (ii) drug toxicity and (iii) novel drug compound effects in humans for predictive purposes, for either short or longer term studies. The generation of a novel tool that improves the predictive power of preclinical efficacy/toxicity studies is of substantial benefit to the health industry as a whole, and will aid in the generation of human-specific target compounds with a significantly higher safety profile.

## Methods

### Microfabrication

The microfabrication of the silicon cantilever for this study was performed at the Cornell NanoScale Science and Technology Facility. All mask designs were created in L-Edit layout editor. The cantilevers were fabricated with a 4 in double sided polished Silicon on insulator (SOI) wafer, with a device thickness of 4 μm, buried oxide thickness of 1 μm and a handle thickness of 500 μm. The device layer of the SOI wafer was spin coated with photoresist and the cantilever front side pattern was defined using standard photolithography techniques. A deep reactive ion etching (DRIE) process was performed on the photoresist patterned device layer to etch through the silicon until the buried oxide layer was visible. The handle layer (backside) of the SOI wafer was spin coated with photoresist and the backside window/cleave lines pattern was defined. A DRIE of the patterned handle layer was performed in multiple runs to achieve a through etch of the handle layer until the buried oxide layer was visible. Following the DRIE step, wet etching of the SOI wafer with 25% dilute HF was performed to strip the buried oxide layer below the cantilevers to release the Si cantilevers and also open a window underneath to provide laser access to probe the cantilevers. The SOI wafer was then rinsed in a DI water bath and oven dried. The fully processed SOI wafer was manually cleaved along etched cleave lines to obtain the individual cantilever chips.

### Cell culture

All human cells types were cultivated at 37 °C with a 5/95% CO_2_/Air mixture in a humidified atmosphere in their own specific medium before being transferred to the co-culture. The different cultures were started at different times in advance of the co-culture integration (liver; 4, cardiac; 7, muscle; 22 and neurons; 35 days). Culture origin, growth, maintenance, differentiation and plating, for the different cell types, is detailed below.

Human hepatocellular carcinoma HepG2/C3A (ATCC^®^ CRL-10741™) were routinely grown, up to fifteen passages, in HepG2/C3A medium (Dulbecco’s Modified Eagle Medium (DMEM) (from Life technologies) supplemented with 10% fetal bovine serum (FBS), 1 mM Sodium Pyruvate (all from Life Technologies) and 5 mM Hepes (Sigma)). Four days (approximately) before transferring to the *in vitro* system, 6 × 10^4^ cells (0.34 cells/mm^2^) were plated in collagen type-I coated 15 mm diameter round glass coverslips. Medium was replaced with HepG2/C3A medium the following day and every 2 days afterwards until co-culture.

Human induced pluripotent stem cell (iPSc) differentiated Cardiomyocytes (Cellular Dynamics International, CDI) were plated according to user instructions and one cryovial was used for each experiment. Cardiomyocytes were plated with CDI cardiac plating medium, 7 days before transferring to the *in vitro* system, on control SiO_2_ surfaces (500 cells/mm^2^), or cantilevers (2,500 cells/mm^2^) previously coated with fibronectin (50 μg/mL in 1X PBS (phosphate buffer solution)). Forty-eight hours later, the medium was replaced with cardiac serum-free maintenance medium^63^, and followed by half medium changes every 48 hours before co-culture.

Human Skeletal Myofibers were obtained as a gift from H. Vandenburg, Brown University without identifiers and prepared by isolation and differentiation steps previously described by this group, with small modifications^64^. Biopsies were performed according to procedures approved by the Institutional Clinical Review Board of the Miriam Hospital^65^. For each culture, human skeletal muscle (hSKM) SCs/progenitors were plated 22 days (approximately) before the co-culture on N-1(3-(trimethoxysilyl) propyl) diethylenetriamine (DETA) modified 15 mm diameter coverslips (30 cells/mm^2^) in hSKM Growth Medium (Lonza), and fed afterwards every 2 days by replacing the medium. On day 7, myoblast fusion into postmitotic myofibers was induced by incubation with differentiation medium 1^64^. The cells were fed every 2 days by changing half of the medium. Four days later, the cells were fed with NBActive4 (Brain Bits) every 2 days by changing half of the medium, for a minimum of 11 days.

Human Motoneurons, obtained from NeuralStem, were differentiated as described^66^ from human spinal cord stem cell line (hSCSC) previously isolated, with slight modifications. Briefly, approximately 35 days before the co-culture, 0.5 × 10^6^ hSCSCs were plated in a T25 flask in Growth medium for 7 days until they were close to confluence. They were then harvested by trypsinization and plated into a PDL/fibronectin-coated 60 mm paranox cell culture dish (Nunc, Cat #174888) at a density of 2.5 million cells/dish and differentiated 4 days in the priming medium followed by 6 days in differentiation medium^67^. Then the cells were replated onto DETA-coated 15 mm diameter round glass coverslips at a density of 250 cells/mm^2^ and cultured in the defined serum-free medium for another 16–18 days before being transferred into the *in vitro* system.

Human iPSc differentiated cortical-like neurons (Cellular Dynamics International, CDI) were plated according to user instructions and one cryovial was used for each experiment. Neurons were plated with CDI neuronal plating medium, 2–4 days before transferring to the *in vitro* system, cells were plated onto DETA modified 15 mm diameter round glass coverslips and then coated with laminin (3.3 μg/mL) at a density of 700 cells/mm^2^. Forty-eight hours later, the medium was replaced with cortical serum-free maintenance medium^68^, followed by half medium changes every 48 hours before the co-culture.

The defined serum-free medium base composition used to maintain the functionality of the 4 organ culture system for up to 14 days was previously published^62^ and was modified by the addition of an antibiotic/antimycotic cocktail at 1X (Life Technologies, 15240-062) and the elimination of the G5 supplement. The base medium was composed of 1X Neurobasal (Life Technologies, 21103-049), 1X B27 (Life Technologies, 17504-044), 1X Antibiotic and antimycotic (Life Technologies, 15240-062) and 1X Glutamax (Life Technologies, 35050-061). It was supplemented with 10 ng/mL Glial-derived Neurotrophic Factor (GDNF) (Cell Sciences, CRG400B), 20 ng/mL Brain-derived Neurotrophic Factor (BDNF) (Cell Sciences, CRB600B), 5 ng/mL Ciliary-derived Neurotrophic Factor (CNTF) (Cell Sciences, CRC400A), 20 ng/mL Neurotrophin-3 and 4 (NT3 and NT4) (Cell Sciences, CRN500B and CRN501B), 100 ng/mL Vitronectin (Sigma, V8379-50UG), 10 ng/mL Insulin-like Growth Factor-I (IGF-1) (PeproTech, 100-11), 100 ng/mL Agrin (R&D, 550-AG-100), 1 μM Adenosine 3′, 5′-cyclic monophosphate (cAMP) (Sigma, A9501), 4 μg/mL laminin (Life Technologies 23017-015), 50 ng/mL Sonic Hedgehog, N-terminal peptide (Shh) (R&D, 1845-SH-025) and 0.1 μM Retinoic acid (Sigma, R2625). All the compounds purchased as a powder were dissolved in water, except for retinoic acid which was dissolved in DMEM 1X.

All experiments utilized cells that were commercially available or derived from commercially available cells. Additional IRB protocols or informed consent at UCF was not necessary as no cell lines contained identifiable information.

### Experimental procedure

The four organ *in vitro* systems were assembled, on day 0, by placing the corresponding surfaces cell side up in a specific chamber of the housing (liver coverslip into chamber 1, cardiac coverslips and cantilevers into chambers 2 and 4, muscle coverslips into chamber 5, and neuron coverslips into chamber 3 and they were then topped with the serum-free co-culture medium (1X Neurobasal Medium, 1X B27, 1X AB/AM, 1X Glutamax (all from Life Technologies)) before locking the system. Right after assembling, the medium was completely replaced with 4 mL of the co-culture defined serum-free medium and the systems placed on a rocking platform inside an incubator. Platforms were checked every day by inspecting for expected cell morphology inside the system and by replacing medium (1.2 mL total volume) from the two reservoirs (30% of the total volume was replaced) with fresh medium. Drug incubation was initiated on day 5 of the co-culture by addition of the drug through the reservoir closer to the liver chamber (no. 1), allowing a first contact of the drug with the liver. 20 μL of the drug stock solution (or vehicle) to achieve a working concentration in 4 mL of total volume in the platform was utilized. On day 6, after 24 hours of drug incubation, 6 μL of the drug stock solution (or vehicle) was added to the fresh medium exchange volume (1.2 mL). After 48 hour of drug incubation, on day 7, systems were disassembled to test organ functionality that same day. For mono-culture conditions, surfaces remained in a 12-well plated with 1 mL of the co-culture defined serum-free medium maintained by daily replacement of half volume.

#### Bright-Field Cell Imaging

Cell morphology and function were studied each day with an inverted bright-field microscope (Carl Zeiss) using a 10X objective. This working distance allowed the imaging of the cells in the specific surfaces inside the housing. Images of each fraction and systems were collected with an Axio Cam and AxioVision AC software to study morphological or functional changes in the co-culture chamber for each organ.

#### Human Serum Albumin quantification

Human Serum Albumin (HSA) production from the liver fraction was tracked throughout the co-culture, by measuring the HSA concentration in the supernatant of the culture as it represents liver protein anabolism. Samples were collected from the exchanged medium (1.2 mL) and stored at −20 °C until analysis. One freeze-thaw cycle occurred before samples were analyzed following manufacturers’ instructions provided in the ELISA Starter Accessory Kit (Bethyl Laboratories Inc, Cat #: E101). Samples were diluted as necessary in sample conjugate/diluent buffer to ensure readings measured within the range of the standard curve. The standard curve was prepared by serial dilutions of human reference serum in sample conjugate/diluent buffer (0–1600 ng/ml). After the reaction was stopped optical densities (OD) were immediately measured at 450 nm using a Synergy HT plate reader and KC4 software.

Final HSA concentrations were calculated in an excel file by first removing the average OD reading measured for blanks from average sample and standard OD readings. Concentrations of standard dilutions were plotted against average OD values measured in XY scatter plots. A standard curve was created by adding an order of 2 polynomial trendline to XY scatter plots and selecting the option to display the equation of the trendline, as well as the R^2^ value, on each scatter plot (for ease of analysis HSA sigmoidal curves were split into 3 standard curves with HSA concentration ranges of 0–200 ng/ml, 200–800 ng/ml and 400–1600 ng/ml). Quadratic equations were rearranged to make X (sample HSA concentration) the unknown variant. Y values (OD values) and a/b/c constants generated from trendline equations were inputted into rearranged quadratic equations per assay. HSA concentrations calculated were corrected accordingly for any differences in culture final volumes, sample dilutions during assays and cells number.

#### Urea quantification

Urea production, corresponding to liver protein catabolism, was analyzed from the same samples as for HSA, thus tracking daily concentration changes. For this colorimetric assay, manufacturers’ instructions provided for the QuantiChromTM Urea Assay Kit (BioAssay Systems, Cat #: DIUR-500) were followed. Plates were incubated in the dark for 50 minutes at room temperature, then OD was measured at 520 nm, using a Synergy HT plate reader and KC4 software. Final urea concentrations were calculated in the same way as for HSA, and plotted as urea produce per day.

#### Cytochrome p450 1A1 and 3A4 enzymatic activity

Enzymatic activities for 1A1 and 3A3 isoforms were assessed in the liver fraction (in mono-culture ± drugs) individually, as an end point evaluation according to the manufacturer’s instructions (p450-610TM assay from Promega) and Li, 2009^64^. Enzymatic products from 1A1 were run with LDR, and separately, 3A4 were run with LDR containing esterases. Enzymatic activities were finally represented as D-Luciferin picomoles produce in 1 hour by 1 × 10^6^ cells.

#### Force measurement

Force measurements were quantified from the cardiac organ chamber after 14 days in co-culture. Individual surfaces were evaluated for spontaneous contraction, or contraction induced by a broad field electrical stimulation on microscale silicon cantilevers as previously reported from this group^4^. Cardiomyocytes were interrogated for spontaneous and stimulated contraction. The resulting force calculations were performed using a modified version of Stoney’s equation^5^.

#### Cardiac beating frequency

Seven or fourteen days after the co-culture, cardiac beating frequency was quantified from video recordings of three different areas of each of the cMEA surfaces, where the cardiomyocytes culture where located. Surfaces were located in a 37 °C bath platform containing co-culture medium with 50 mM HEPES on the stage of a Zeiss Axioscope 2FS upright microscope. Beats were counted in a period of 10 s, and the average number of 3 values was obtained. Drug effect was plotted as percentage of change compared to the control conditions.

#### Myotube stimulated contraction

Myotube contraction in coverslips was evaluated after the co-culture was finalized. Individual surfaces were evaluated as previously reported in the group^6^. Drugs effects were quantify in a binary code mode, cells were or were not contracting. Representing videos demonstrating contraction synchronously with the stimulation pulse are available as supplementary files.

#### Neuron electrophysiology recording

Electrophysiological properties of neuron cultures where recorded on the last day of co-culture following a previously described method^2^. Values of firing, repetitive firing, spontaneous firing, inward sodium current, outward potassium current, action potential peak voltage and the resting membrane potential, characterized the mature functionality of neurons in the *in vitro* system. Drug alterations of the neuronal electrophysiology are discussed in this manuscript.

#### Immunocytochemistry

Surfaces were fixed in freshly prepared 4% PFA (paraformaldehyde) for 15 minutes after co-culture. Cells were rinsed afterwards, with 1X PBS, 3 times with longer incubations, 5, 10 and 15 minutes at RT. To permeabilize the cells a 0.2% triton X-100 in 1X PBS was incubated for 15 minutes. Non-specific binding sites were blocked with 5% DS and 0.5% BSA in 1X PBS for 1 hour at RT. Primary antibodies were incubated on the cells OVN at 4 °C. The following day, primary antibodies were removed and surfaces were rinsed, with 1X PBS, 3 times. Then, the cells were incubated with secondary antibodies, in the dark, for 2 hours at RT. Surfaces were newly rinsed and incubated with 3 mM 4′-6-Diamidino-2-Phenylindole (DAPI) in 1X PBS for 10 min, in the dark and at RT for nuclei staining. Finally, two more 1X PBS rinses were done before imaging. DAPI incubation was replaced by a Hard Set Mounting with DAPI (Vector laboratories, Inc.) when the cells were on coverslips. Images were collected with Axioskop 2 mot plus upright spinning disk confocal microscope (Carl Zeiss) with a XCite 120 Fluorescence Illumination system (EXFO) beam and a multi-spectral laser scanning, coupled to Volocity software (Perkin Elmer).

The following primary antibodies were used for this study: mouse anti-Troponin (MAB1693, 1:200), and rabbit anti-neurofilament (AB1981, 1:2000) (from Millipore); mouse anti-Myosin Heavy Chain (A4.1025-5 5ea, 1:10) and sheep anti-Human Serum Albumin (ab8940, 1:100) (from ABcam). Secondary antibodies and actin stain used in the secondary antibody cocktail dilutions: donkey anti-mouse 488 (A-21202, 1:200), donkey anti-rabbit 488 (A21206, 1:200), donkey anti-sheep 568 (A21099, 1:200) and Phalloidin 594 (A12380, 1:40) (from Life Technologies).

#### Cell viability

Seven days after the initiation of the co-culture, viability of each organ chamber, except for neurons, was evaluated through an MTT assay. After functional assays were performed, 1.2 mM 3-(4,5-dimethylthiazol-2-yl)- 2,5- diphenyltetrazolium bromide (MTT) (Sigma-Aldrich) diluted in culture medium was incubated for 2 hours at 37 °C. Supernatant removal was followed by the addition of the solubilization reagent (0.57% acetic acid and 1% SDS in DMSO). Cell viability was measured at 570 nm in a Synergy HT plate reader and KC4 software. The results were expressed as the percentage of cell survival relative to the control (untreated or incubated with vehicle). The neuronal fraction viability was evaluated through morphological analysis throughout the co-culture experiment as the final coverslips were used for patch-clamp electrophysiology. Phase images (10X) where obtained every day of the co-culture. Neuronal viability was evaluated by cell morphology integrity and by cell count from day 1 to day 14.

### Statistical methods

Values are expressed as the mean ± SE of a minimum of three independent experiments. Data were evaluated by unpaired Student’s t-test when analyzing the trend of change between two conditions, control and drug-treated. Student’s t-test analysis was run with a one tail distribution and a two sample unequal variance. Cardiac beating frequency data was transformed (applying a logarithm base 10) to reduce the variance between treatments, and then analyzed with the t-test. One-way ANOVA test was used for different conditions that differ in one parameter (i.e. time). Both analysis were ran with Microsoft Excel Statistical tool. Differences with p-values <0.1 were taken as statistically significant.

### Materials

Doxorubicin (DOX), Valproic Acid (VPA), Acetaminophen (APAP), 3-Acetamidophenol (AMAP) and Atorvastatin calcium (ATR), Dimethyl Sulfoxide (DMSO), Bovine Serum Albumin (BSA) (all purchased from Sigma). PDMS, Paraformaldehyde (PFA) (Electron Microscope Sciences), Donkey Serum (DS) (S30, Millipore), Collagen type-I from Rat tail (A10483-01, Gibco) was diluted to 60 μL/mL with 0.02 M Acetic acid (Sigma) in 1X PBS, and Fibronectin (FC010, Millipore) was diluted to 50 μg/mL in 1X PBS. Laminin was diluted to 3.3–4 μg/mL in mQ water.

### Drug Concentrations and stock solutions

Drug stock solutions were prepared taking into account drug solubility and final concentrations targeted. Doxorubicin (1 mM) and Valproic Acid (400 mM) were reconstituted in purified water. Acetaminophen (4 M), 3-Acetamidophenol (4 M) and Atorvastatin calcium (20 mM) were reconstituted in DMSO. Stock aliquots were stored at −20 °C until needed. Acetaminophen and 3-Acetamidophenol were further diluted with media, previous to cell treatment, to a 1 M solution (<0.13% DMSO). Vehicle control was used as a control for Atorvastatin treatment (0.5% DMSO).

## Additional Information

**How to cite this article**: Oleaga, C. *et al*. Multi-Organ toxicity demonstration in a functional human *in vitro* system composed of four organs. *Sci. Rep*. **6**, 20030; doi: 10.1038/srep20030 (2016).

## Supplementary Material

Supplementary Information

Supplementary Movie S1

## Figures and Tables

**Table 1:** Cytotoxic effects of Doxorubicin, Atorvastatin, Valproic Acid, Acetaminophen and N-Acetyl-m-aminophenol.

	Liver		Muscle		Neurons		Cardiomyocytes	
	Literature	4-organ system	Literature	4-organ system	Literature	4-organ system	Literature	4-organ system
Drug Results-Doxorubicin								
Drug effects on cell viability	Toxicity reported	49 ± 10% loss	Toxicity at higher concentrations	Toxicity not observed	Neurologic disturbances (**)	33 ± 13% loss of cell viability	Toxicity reported	65 ± 6% loss
Drug effects on cell functionality	May be compromised	HSA and Urea not affected(*)		60% loss of contractile ability		Electrophysiology affected	Compromised	47 ± 9% decrease in beating rate
Drug Results-Atorvastatin								
Drug effects on cell viability	Toxicity reported	50 ± 16% loss	Toxicity reported	30 ± 15% loss	Toxic at higher concentration(**)	47 ± 14% loss of cell viability	Cardio-protective effect	No toxic effect observed, 24 ± 8% increase in viability
Drug effects on cell functionality	May be compromised	24 ± 4% increase in Urea, HSA not affected(*)	Compromised	50% loss of contractile ability		Electrophysiology affected		43 ± 16% increase in beating rate
Drug Results – Valproic Acid								
Drug effects on cell viability	Toxicity reported	56 ± 16% loss	Toxicity not reported	Toxicity not observed	Toxicity not reported	No toxic effect observed, some increase in cell numbers but not significant	Cardio-protective effect	No toxic effect observed, 36 ± 18% increase in viability
Drug effects on cell functionality	Compromised	13 ± 2% decrease in Urea, HSA not affected(*)		76% loss of contractile ability		Normal electrophysiology		38 ± 18% increase in beating frequency
Drug Results-Acetaminophen								
Drug effects on cell viability	Toxicity reported	37 ± 9% loss	Myo-protective effect	Toxicity not observed	Toxicity not reported	50 ± 18% loss of cell viability	Cardio-protective effect	Toxicity not observed
Drug effects on cell functionality	Compromised	52 ± 13% increase in Urea, HSA not affected(*)		37% loss of contractile ability		Electrophysiology affected		27 ± 9% increase in beating frequency
Drug Results – N-Acetyl-m-aminophenol								
Drug effects on cell viability	Toxicity not reported	Toxicity not observed	Toxicity not reported	Toxicity not observed	Toxicity not reported	Toxicity not observed, some increase in cell numbers but not significant	Toxicity not reported	28 ± 6% loss
Drug effects on cell functionality		138 ± 19% increase in Urea, HSA increased 23 ± 4%(*)		No loss in contractile ability		Normal electrophysiology		25 ± 4% decrease in beating rate

**Doxorubicin**. (*) In liver monocultures, longer incubations (120 hours) with doxorubicin (5 μM) causes a decrease in albumin production over time with no effect in urea production (data not shown). (**) Neurologic disturbances as a result of cognitive impairment during long term chemotherapy treatments. **Atorvastatin**. (*) In liver monocultures, longer incubations (120 hours) with atorvastatin (100 μM) induced urea production (data not shown). (**) No conclusive data, neurotoxicity and neuroprotection effects are claimed based on statin concentration. **Valproic Acid**. (*) In liver monocultures, longer incubations (96 hours) with valproic acid (2 mM) induced urea production (data not shown). **Acetaminophen**. (*) In liver monocultures, longer incubations (96 hours) with acetaminophen (5 mM) did not affect urea production and increased albumin (data not shown).
