# Supplementary material for: Multi-Organ toxicity demonstration in a functional human in vitro system composed of four organs
Source: Sci Rep. 2016 Feb 3;6:20030. doi: 10.1038/srep20030 (PMC4738272; doi:10.1038/srep20030)
Supplement: Supplementary Information [file srep20030-s1.pdf]

Multi-Organ toxicity demonstration in a functional human *in vitro* system composed of four organs

Authors: Carlota Oleaga[1,†], Catia Bernabini[1,†], Alec S.T. Smith[1], Balaji Srinivasan[1], Max Jackson[1], William McLamb[1], Vivien Platt[1], Richard Bridges[1], Yunquing Cai [1], Navaneetha Santhanam[1], Bonnie Berry[1], Sarah Najjar[1], Nesar Akanda[1], Xiufang Guo[1], Candace Martin[1], Gail Ekman[1], Mandy B. Esch[2], Jessica Langer[3], Gladys Ouedraogo[4], Jose Cotovio[4], Lionel Breton[4], Michael L. Shuler[2] and James J. Hickman[1,*]

Supplementary

Simulation and modeling (CFD Simulation)

In the present study, CFD-ACE+, a commercially available computational fluid dynamics(CFD) package was applied to perform a transient simulation of the gravity driven flow through the microfluidic system placed on a rocker platform. The simulation results provided time dependent flow rates and shear stress distribution within the microfluidic system for the selected operating conditions of the rocker platform. CFD-GEOM was applied to define the geometry and generate a structured mesh throughout the liquid volume. Supplementary Figure 1A shows the isometric view of the generated structured mesh.

The flow module in CFD-ACE-GUI was selected to solve for velocity vector, total pressure and wall shear stress. A no slip boundary condition was applied on all walls and a fixed pressure boundary condition with atmospheric pressure was applied at the inlet/outlet of the model. The model was setup to solve for transient solution with time step of 1 second and number of time steps corresponding to the oscillation time period of the rocker platform. A user subroutine was coded in FORTRAN to define the gravity body force components as the driving forces for the fluid flow within the model. The tilt angle and time period of oscillation were defined as variables to set the operating conditions of the rocker platform. The initial conditions for the model were set to zero for both velocity and constant pressure. The spatial differencing method for velocity was set to first order Upwind method. The default linear solver types,

namely Conjugate Gradient Squared (CGS)[64] and Algebraic Mutligrid (AMG)[64], were applied for velocity and pressure respectively. The inertial and linear relaxation values were set to default values.

The model was solved for two operating conditions of the rocker platform, namely 2 degrees tilt with 1 oscillation per minute and 8 degrees tilt with 2 oscillations per minute. CFD-VIEW was used to view the simulation results and perform post-processing of the simulation data. Supplementary Figure 1B shows the solved model with arrows representing the velocity vectors. Supplementary Figure 1C illustrates the change in flow direction (represented by velocity vectors) for the bi-directional flow within the interconnecting channels at two time instances corresponding to change in tilt direction of the rocker platform. The shear stresses on the floor of each chamber were plotted as surface plots. Supplementary Figure 1D shows a typical shear stress surface plot indicating high and low regions of shear stress.

It can be observed that the shear stress regions are confined to the inlet and outlet of the chamber due to the smaller widths of the inlet/outlet interconnecting microchannels. The shear stress is also comparatively higher in the narrower chambers (chambers 4 and 5) when compared to the wider chambers (chamber 2 and 3). The centerline velocity and peak shear stress values were compared for the two operating conditions of the rocker platform. Supplementary Figure 1E shows a comparison of shear stress on the floors of the chambers for the two operating conditions of the rocker platform. Supplementary Figure 1F shows a comparison of centerline velocity within the device for the two operating conditions of the rocker platform. Both the shear stress and centerline velocity values were observed to increase as the tilt angle of the rocker platform was increased from 2 degrees to 8 degrees. The increase in number of oscillations per minute (opm) of the rocker platform also increases the shear stresses and center line velocity. The chambers 3 and 5 are not indicated in Supplementary Figure 1E-F since they are symmetrical to chambers 2, 4 and will have identical results.

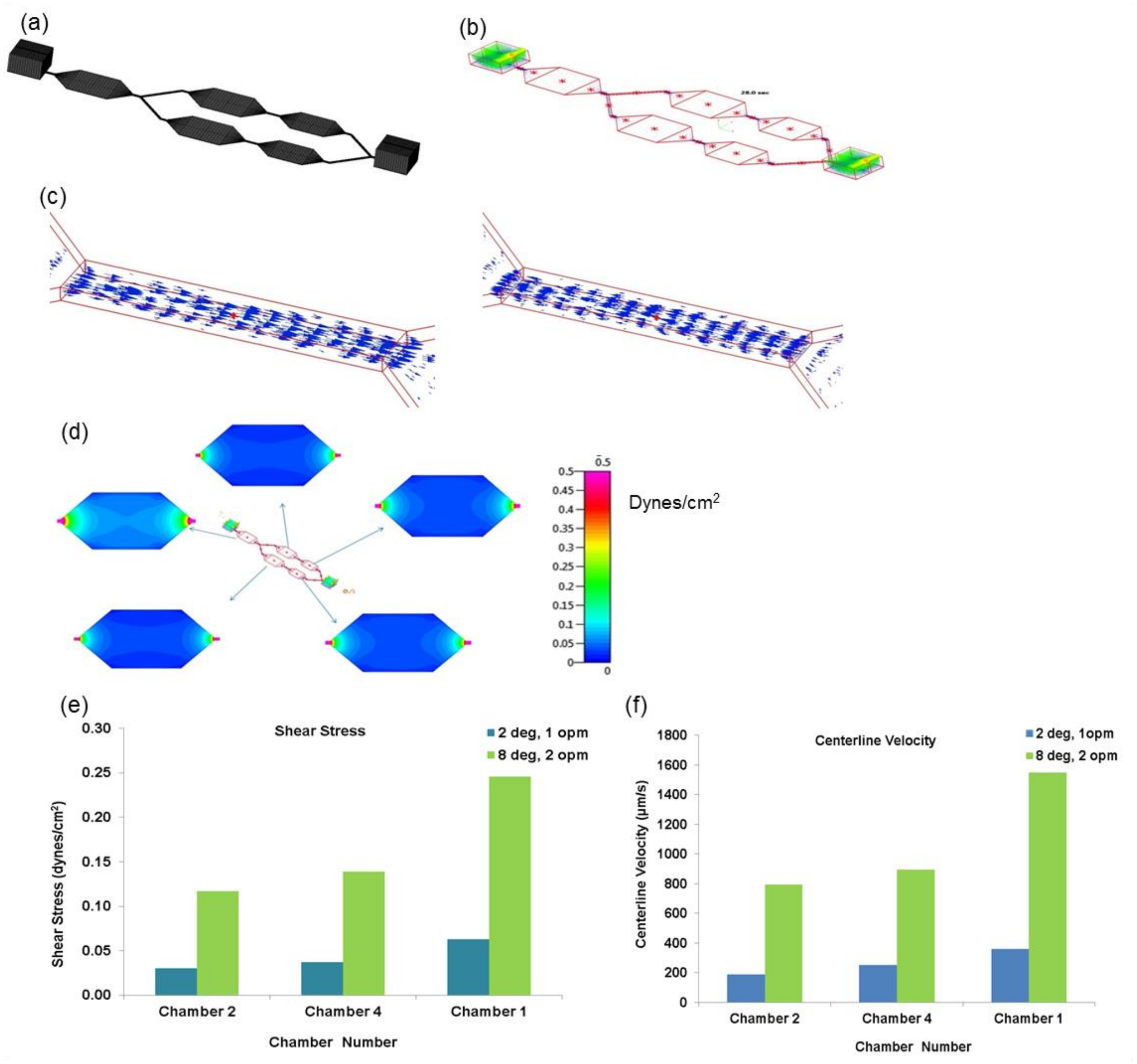

Supplementary Figure 1. (a) Isometric view of the structured mesh **(b)** Solved model illustrating the velocity vectors at t = 28 s **(c)** A bi-directional gravity driven flow (with arrows representing velocity vectors) within the interconnecting microchannel between two chambers of the device Flow direction to right at t = 15s and flow direction to left at t = 45s **(d)** Surface plots indicating the shear stresses (scale: 0 - 0.5 dynes/cm^2) at the floor of each chamber of the device **(e)** Comparison of shear stress at the floor of chambers 2, 4 and 1 for the two operating conditions of the rocker platform **(f)** Comparison of centerline velocity within the chambers 2, 4 and 1 for the two operating conditions of the rocker platform.

Supplementary Video 1. Functional data of skeletal muscle contractility was assessed by video analysis after 14 days in the 4 organ system under flow.
